# Bi-regional training course for staff managing healthcare-assocaited infection (HAI) with minimal resources

**DOI:** 10.1186/1753-6561-5-S6-P284

**Published:** 2011-06-29

**Authors:** PP-L Or, E Chan, D Tong, M Chow, P Ching, W Seto, S Pang, K Fritsch

**Affiliations:** 1 School of Nursing, The Hong Kong Polytechnoc University, Hong Kong, Hong Kong, China; 2 Nursing Division, Hong Kong, Hong Kong, China; 3 Infectious Disease Centre, Hong Kong, Hong Kong, China; 4 World Health Organization Collaborating Centre for Infection Control, Hospital Authority, Hong Kong, Hong Kong, China; 5 Western Pacific Region, World Health Organization, Manila, Philippines

## Introduction / objectives

To prevent and control Healthcare-Associated Infection (HAI), it is necessary to have adequately trained staff and substantial financial resources. The aim of this training was to train personnel at various levels in the healthcare system in multifaceted infection control practices specifically for situations where resources are limited.

## Methods

The training programme consisted of lectures, interactive group work using the PPRR (Prevention, Preparedness, Response and Recovery) model on infectious diseases, a skills test on Personal Protective Equipment (PPE), and a written test. Each participant was required to complete the assessment checklist and an action plan for developing effective infection control in their countries.

## Results

69 health care professionals from 16 countries were trained. In their home countries, 37 (53.6%) worked in hospitals; 31 (44.5%) worked in Ministry of Health agencies, and 6 (8.7%) were on teaching faculties of universities. The highest score on the written test was 46 out of 50 and the lowest score was 27 out of 50. All the participants could answer two questions correctly about urinary catheter. Participants strongly agreed that the course was relevant; they were satisfied with the training method.

## Conclusion

We will further consolidate the training material into a toolkit and establish a network with our course participants in order to support and facilitate their work in their home countries. Infection Control Elluminate is held every two weeks to share and review their work.

## Disclosure of interest

None declared.

